# The use of specialised preterm birth clinics for women at high risk of spontaneous preterm birth: a systematic review

**DOI:** 10.1186/s12884-020-2731-7

**Published:** 2020-01-29

**Authors:** Lisa Dawes, Katie Groom, Vanessa Jordan, Jason Waugh

**Affiliations:** 1 Liggins Institute, The University of Auckland, Private Bag 92019, Victoria Street West, Auckland, 1142 New Zealand; 2 National Women’s Health, Auckland City Hospital, Auckland, New Zealand; 3 Department of Obstetrics & Gynaecology, The University of Auckland, Auckland, New Zealand; 4 Cochrane New Zealand, Auckland, New Zealand

**Keywords:** Specialised preterm birth clinic, Preterm birth prevention clinic, Spontaneous preterm birth

## Abstract

**Background:**

Specialised preterm birth clinics care for women at high risk of spontaneous preterm birth. This systematic review assesses current practice within preterm birth clinics globally.

**Methods:**

A comprehensive search strategy was used to identify all studies on preterm birth clinics on the MEDLINE, Embase, PsycINFO, CENTRAL and CINAHL databases. There were no restrictions to study design. Studies were limited to the English language and publications from 1998 onwards. Two reviewers assessed studies for inclusion, performed data extraction and reviewed methodological quality. Primary outcomes were referral criteria, investigations and interventions offered in preterm birth clinics. Secondary outcomes were the timing of planned first and last appointments and frequency of review.

**Results:**

Thirty-two records fulfilled eligibility criteria and 20 studies were included in the main analysis following grouping of records describing the same study or clinic. Studies were of mixed study design and methodological quality. A total of 39 clinics were described; outcome data was not available for all clinics. Referral criteria included previous spontaneous preterm birth (38/38, 100%), previous mid-trimester loss (34/38, 89%) and previous cervical surgery (33/38, 87%). All clinics offered transvaginal cervical length scans. Additional investigations varied, including urogenital swabs (16/28, 57%) and fetal fibronectin (8/28, 29%). The primary treatment of choice for a sonographic short cervix was cervical cerclage in 10/33 (30%) clinics and vaginal progesterone in 6/33 (18%), with 10/33 (30%) using multiple first-line options and 6/33 (18%) using a combination of treatments. The majority of clinics planned timing of first review for 12–16 weeks (30/35, 86%) and the frequency of review was usually determined by clinical findings (18/24, 75%). There was a wide variation in gestational age at clinic discharge between 24 and 37 weeks.

**Conclusions:**

There is variation in the referral criteria, investigations and interventions offered in preterm birth clinics and in the timing and frequency of review. Consistency in practice may improve with the introduction of consensus guidelines and national preterm birth prevention programmes.

**Trial registration:**

*Systematic review registration number*: CRD42019131470.

## Introduction

Preterm birth is the leading cause of neonatal death and is associated with significant perinatal morbidity and lifelong health consequences [1]. Preterm birth is common and accounts for approximately 10% of births worldwide [1]. At least half of all preterm births are the result of spontaneous onset of labour or pre-labour rupture of membranes [2]. Despite considerable research efforts there is no effective treatment to stop preterm labour once it has established and current management focuses on prevention [3, 4]. In recent years, specialised preterm birth clinics have developed due to a growing understanding of risk factors for preterm birth and the importance of risk stratification to guide the use of interventions to prevent preterm labour [5]. To the best of our knowledge, the first modern-day preterm birth clinic was established in the United Kingdom (UK) in 1998.

Preterm birth clinics provide focused and specialised obstetric care to asymptomatic women at increased risk of preterm birth due to their obstetric or gynaecological history. The key components include addressing modifiable risk factors (such as advice on becoming smoke-free, and screening and treating infection), surveillance of cervical length by transvaginal ultrasound scan through the mid-trimester, and providing evidence-based interventions when indicated. The use of transvaginal cervical length assessment and quantitative fetal fibronectin have been proven to aid prediction of spontaneous preterm birth in asymptomatic high risk women and can be used to guide management decisions [6, 7]. Interventions such as vaginal progesterone and cervical cerclage have been shown to reduce spontaneous preterm birth and associated neonatal morbidity in asymptomatic, high risk women who develop a sonographic short cervix in the mid-trimester [8–10].

Although there is good evidence to support many of the practices that occur in preterm birth clinics, specific evidence to support the utility of preterm birth clinics as a whole is still evolving [5]. Two previous systematic reviews have attempted to assess the efficacy of preterm birth clinics in reducing spontaneous preterm birth and improving neonatal outcomes [11, 12]. Neither found conclusive evidence to either support or refute the efficacy of specialised preterm birth clinics compared to standard antenatal care [11, 12]. However, both acknowledged the limited number of studies in this field; only five were randomised controlled trials, all of which were conducted prior to 1990 and no longer reflect practice in modern-day preterm birth clinics. It is unlikely that further randomised controlled trials will be carried out due to the multi-faceted and complex nature of the intervention [5]. Despite the lack of direct evidence to support the use of preterm birth clinics, the poor outcomes from preterm birth, the availability of multiple evidenced-based interventions, and the ability to provide coordinated and individualised care provide sufficient justification for resourcing these clinics [5]. Preterm birth clinics have become standard care in many countries and are recommended in the UK [13].

### Rationale

Until recently there were no national or international guidelines on the protocols and care pathways to be used in preterm birth clinics, and practice is often based on local expert opinion. The newly released (2019) ‘Reducing Preterm Birth: Guidelines for Commissioners and Providers’ from the UK Preterm Clinical Network provides guidance on referral pathways for preterm birth prevention [13]. This includes recommendations on timing and frequency of cervical length assessments and use of quantitative fetal fibronectin testing, along with management options including cervical cerclage, progesterone and cervical pessary, with reference to the National Institute for Health and Care Excellence (NICE) Guidelines for preterm birth [13, 14].

This systematic review aims to assess the referral criteria and investigations and interventions offered in preterm birth clinics internationally and the planned timing and frequency of review. It does not attempt to prove the efficacy of preterm clinics as it has already been established that there is currently inadequate evidence available [11, 12]. The results of this systematic review will be useful for future work in improving consistency in care in both established and new preterm birth clinics. This will in turn allow results from future high-quality observational studies to be more accurately synthesised in systematic review and meta-analyses to assess the efficacy of preterm birth clinics in reducing spontaneous preterm birth and improving offspring outcome.

### Objectives

This systematic review has four objectives:

1. To assess the eligibility criteria used for referral to preterm birth clinics.
2. To assess the types of investigations offered in preterm birth clinics.
3. To assess the types of interventions offered in preterm birth clinics.
4. To assess the planned frequency and timing of review in preterm birth clinics.

## Methods

### Protocol and registration

The protocol was prospectively registered with the PROSPERO International Prospective Register of Systematic Reviews in May 2019, registration number CRD24019131470, available at https://www.crd.york.ac.uk/prospero/display_record.php?RecordID=131470. This systematic review has been conducted in line with the standard Preferred Reporting Items for Systematic Reviews and Meta-Analyses (PRISMA) statement and written according to the PRISMA checklist of items to include when reporting a systematic review [15].

### Eligibility criteria

All studies on preterm birth clinics were eligible for inclusion, including those that assessed a clinic indirectly i.e. by assessing another intervention in a high risk population cared for in a preterm birth clinic. Preterm birth clinics are also known as preterm birth prevention clinics, preterm surveillance clinics, specialised preterm birth clinics, dedicated preterm birth clinics, miscarriage follow-up clinics and specialised antenatal clinics. There is no comparator group in this review due to the nature of the research objectives. Studies on other types of specialised antenatal clinics such as for multiple pregnancy, hypertension and diabetes were excluded.

There were no restrictions placed on the types of studies eligible for inclusion and both quantitative and qualitative research methods were included. Examples of study designs include randomised controlled trials, cohort studies, case-controlled studies, cross-sectional studies, interviews, surveys and focus groups. Studies were restricted to those published in the English language, for feasibility, and to publications from 1998 onwards, as this is when the first modern-day preterm birth clinic was established.

The primary outcome measures are:

1. Eligibility criteria for referral (for example, previous spontaneous preterm birth prior to a specified gestation, previous cervical surgery of specific type or depth of excision).
2. Types of investigations offered, defined as any test arranged or carried out from the clinic with the aim of reducing the risk of spontaneous preterm birth or improving perinatal outcomes from preterm birth (for example, urine culture, urogenital swabs, cervical length ultrasound, fetal fibronectin). Investigations that form part of standard antenatal care that are not aimed at reducing the risk of spontaneous preterm birth were excluded (for example, aneuploidy screening, fetal anatomy scan).
3. Types of interventions offered, defined as any surgical, medical or non-medical therapy used with the aim of reducing the risk of spontaneous preterm birth (for example, cervical cerclage, progesterone, cervical pessary) or with the aim of improving perinatal outcomes for babies that are born preterm (for example, antenatal corticosteroids, hospital admission).

The secondary outcomes measures are:

4. Timing of planned first and last clinic visit (measured in weeks and days of gestation).
5. Frequency of planned clinic review (measured in number of days or weeks).

### Information sources

The MEDLINE, Embase, PsycINFO, CENTRAL and CINAHL databases were searched on 1 May 2019. Additional studies were identified by hand-searching reference lists of included publications.

### Search

A comprehensive search strategy was developed using the Peer Review of Electronic Search Strategies (PRESS) Guidelines [16] and was adapted for each of the five databases. The search strategy utilised keyword terms for a preterm birth clinic, and MeSH terms for outpatient pregnancy care combined with MeSH terms for preterm birth, pregnancy complications or high risk pregnancy. A human filter was applied along with limits for the English language and for references published from 1998 onwards. The MEDLINE search is available in Additional file 1: Table S1.

### Study selection

References identified from each database search were imported into EndNote X8 referencing software [17] and then into Covidence systematic review software [18]. Duplicates were identified and excluded. References were screened independently by two reviewers for potential eligibility based on the title and abstract. Full-text articles were retrieved for references that appeared to be relevant and these were also independently assessed for inclusion by two reviewers. Discrepancies were resolved through discussion. Records were combined if they described the same study, e.g. conference abstracts with full-text articles; and studies that had been updated. For updated studies, the most recent record was used as the study identifier to describe both the original and updated study, and was used for the majority of data collection. Studies were also grouped when there was more than one study describing an individual clinic, with the most relevant study selected following discussion between two investigators. This selected study was used as the study identifier and for the majority of data collection, with the additional studies used for missing data. This approach was necessary to prevent over-representation of clinics that were described in more than one study. All studies that reported on multiple (named or unnamed) clinics were included at this stage for simplicity and over-representation was addressed later in synthesis.

### Data collection process

Electronic data collection forms were used to extract and record data from included studies. Data collection was performed by one reviewer and cross-checked by a second reviewer. Authors were contacted for the names and locations of included preterm birth clinics when this was not reported.

### Data items

Primary and secondary outcomes previously specified were collected. Other data items include study source information and funding details, study design, study timeframes, demographic details, risk factors for spontaneous preterm birth, and spontaneous preterm birth rates.

### Risk of bias and quality assessments

Two reviewers assessed the methodological quality of included studies. The Cochrane Risk of Bias Tool [19] was used for randomised controlled trials, the Newcastle-Ottawa Scale [20] for cohort, case controlled studies and other observational studies, the modified Newcastle-Ottawa Scale [21] for cross-sectional studies and the Critical Appraisal Skills Programme (CASP) Checklist [22] for qualitative studies.

### Summary measures

Primary and secondary outcomes are described as proportions.

### Synthesis of results

The majority of studies included UK based preterm birth clinics, and some reported on multiple clinics. To ensure we avoided over-representation of clinics described in more than one study, authors of UK studies that reported on unnamed clinics were approached [23–26]. This allowed us to assess whether the largest and most comprehensive study on preterm birth clinics (Care 2019) [23] included all UK clinics described in other studies. All but four clinics in the Care 2019 study were identified and alternative studies including them were excluded from synthesis [25–35]. Of the four clinics reported anonymously in the Care 2019 study, three are also believed to have been reported elsewhere and so these studies were also excluded [24, 36]. Data from Care 2019 were amalgamated with data from remaining studies, all of which reported on individual clinics outside of the UK, to provide an overall synthesis for the primary and secondary outcomes. A narrative synthesis is provided, structured around the outcome measures, with information also presented in tables. No meta-analysis was performed.

## Results

### Study selection

**Fig. 1:**
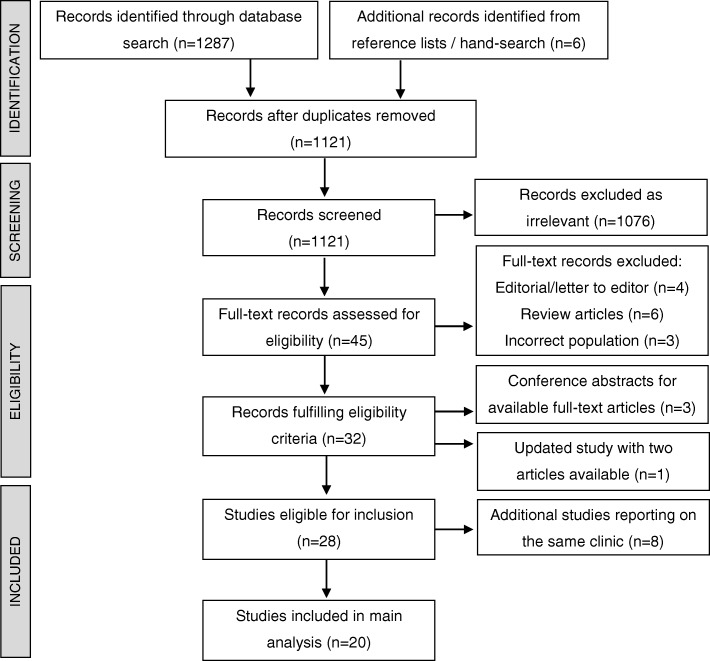
PRISMA flow chart of study selection

The study selection process is detailed in Fig. 1. Of the 1293 records identified from the search strategy, 32 fulfilled eligibility criteria. Three of these were conference abstracts for included full text articles [37–39]. One study had been updated and the two publications were combined [23, 40]. A further eight were additional studies reporting on individual clinics already represented by another included study [41–48] (detailed in Table 1). Twenty studies were therefore included in the main analysis.

**Table 1:** Grouping of studies when more than one study reports on an individual clinic

Main study	Additional studies on the same clinic	Name and location of preterm birth clinic
Bolt 2011	Khambay 2012 Duhig 2009 Min 2016 Smout 2010^a^	Guys and St Thomas’ Hospital, London, United Kingdom
Ivandic 2018	Alfirevic 2013 Care AG 2014 Care A 2014^a^	Liverpool Women’s Hospital, Liverpool, United Kingdom
Karkhanis 2012^a^	Raouf 2009^a^	Birmingham Heartlands Hospital, Birmingham, United Kingdom

^a^ Conference abstract only

### Study characteristics

Of the 20 included studies, 15 were full text articles [23, 24, 27–29, 33, 34, 49–56] and five were conference abstracts [26, 30–32, 36]. Fourteen studies reported on an individual clinic [27–33, 36, 49–53, 56] and six studies reported on multiple clinics [23, 24, 26, 34, 54, 55]. There were a variety of study designs; seven retrospective audits [26, 28–32, 51], three prospective observational studies [24, 36, 55], two cross-sectional studies [23, 49], two prospective cohort studies [54, 56], two retrospective cohort studies [50, 52], two other retrospective observational studies [27, 34], one randomised controlled trial [53], and one qualitative interpretive study [33]. A total of 39 clinics were assessed in data synthesis; 33 clinics (87%) were in the UK, two in America, two in Australia, and one each in Germany and Italy.

### Risk of bias and quality assessments

Results of the methodological quality assessments are shown in Additional file 1: Tables S2-S5. Methodological quality was mixed with studies of low, medium and high quality.

### Results of individual studies

The study characteristics and primary and secondary outcomes are summarised in Table 2 for studies on individual clinics, and in Table 3 for studies reporting on multiple clinics.

**Table 2:** Characteristics of included studies reporting on an individual clinic

Study details	Characteristics of women cared for in the clinic and spontaneous preterm birth rate	Eligibility criteria for referral to the clinic	Investigations and interventions offered	Timing and frequency of review
1. **Bolt 2011** Retrospective observational study. 12 month period, dates not reported. Guys and St Thomas’ Hospital, London, UK. *Primary and secondary outcome data also obtained from Min 2016, Khambay 2012 and Duhig 2009.*	147 women; White 45%, Black 44%, Asian 4%, other 5% and unknown 3%. Risk factors for spontaneous preterm birth: previous preterm birth > 24 weeks 37%, previous late miscarriage 35%, LLETZ 13%, cone biopsy 11%, previous cerclage 16%, current cerclage 27%. Spontaneous preterm birth rate < 37 weeks 18%.	Previous preterm birth < 37 weeks. Previous PPROM < 37 weeks. Previous late miscarriage 16–24 weeks. Extensive cervical surgery e.g. LLETZ, cone biopsy, trachelectomy. Uterine abnormality. Cervical length < 25 mm on transvaginal scan. Previous cervical cerclage.	*Investigations:* - Transvaginal cervical length ultrasound scans. - Quantitative fFN at 22–30 weeks. *Interventions:* Cervical cerclage: offered electively if ≥ 3 previous spontaneous deliveries or losses at 16–34 weeks or ≥ 2 with an additional risk factor, a previous failed cerclage, or as an ultrasound-indicated procedure if cervical length is < 20 mm on transvaginal ultrasound. If considered high risk based on cervical length and fFN results at 23–28 weeks: - Hospital admission. - Antibiotics if infection suspected. - Tocolysis (nifedipine) if contracting. - Betamethasone.	Timing of planned first visit not reported. Women are seen 2–6 weekly, individualised to clinical need. Women assessed as low risk are discharged at 22–24 weeks. High risk woman are seen up to 30 weeks.
2. **Ivandic 2018** Retrospective audit. January 2013 – December 2017. Liverpool Women’s Hospital, Liverpool, UK. *Primary and secondary outcome data also obtained from Care AG 2014 and Alfirevic 2013.*	129 women; White 85%, Black African 3%, Asian 2%, Arab 3% and other 7%. Risk factors for spontaneous preterm birth: previous preterm birth or PPROM < 34 weeks 76%, LLETZ 21%, knife cone biopsy 9%. Spontaneous preterm birth rate < 37 weeks 50%, < 34 weeks 29%.	Previous spontaneous preterm birth at 16–34 weeks. Previous PPROM at 16–34 weeks. Significant cervical surgery defined as ≥ 2 LLETZ or knife cone biopsy. Uterine abnormalities. Incidental finding of short cervix on ultrasound. Following an episode of threatened preterm labour.	*Investigations:* - Transvaginal cervical length ultrasound scans. *Interventions:* Treatment is offered if transvaginal cervical length < 3rd centile for gestational age, including: - Cervical pessary. - Vaginal progesterone. - Cervical cerclage.	The first visit is planned for 16 weeks, or earlier if there is a history of significant cervical surgery or cerclage in a previous pregnancy. Women are seen 1–4 weekly depending on the initial cervical length and the gestational age of previous preterm births. Women are discharged at 28 weeks.
3. **Karkhanis 2012** Retrospective audit. November 2007 – November 2009. Birmingham Heartlands Hospital, Birmingham, UK. *Primary and secondary outcome data also obtained from Raouf 2009.*	180 women; ethnicity not reported. Risk factors for spontaneous preterm birth: previous preterm labour or mid-trimester loss 88%. Spontaneous preterm birth rate < 37 weeks 32%.	Previous preterm birth. Previous mid-trimester loss.	*Investigations:* - Transvaginal cervical length ultrasound scans. - Midstream urine. - Low vaginal swabs. *Interventions:* - Cervical cerclage. - Vaginal progesterone. - Combined treatment.	The first visit is planned for 16 weeks. Frequency of review is not reported. Women are discharged at 34 weeks.
4. **Yulia 2015** Retrospective audit (conference abstract only). January 2011 – December 2013. Chelsea and Westminster Hospital, London, UK.	63 women; ethnicity not reported. Risk factors for spontaneous preterm birth: previous late miscarriage or preterm delivery < 34 weeks 40%, previous deep cervical treatment 43%, uterine anomaly 8%. Spontaneous preterm birth rate < 37 weeks 16%.	Previous preterm delivery < 34 weeks. Previous late miscarriage. Previous deep cervical treatment. Uterine anomalies.	*Investigations:* - Transvaginal cervical length ultrasound scans. *Interventions:* - Cervical cerclage.	Not reported.
5. **Kindinger 2013** Retrospective audit (conference abstract only). January 2011 – January 2013. St Mary’s Hospital, London, UK.	160 women; ethnicity not reported. Risk factors for spontaneous preterm birth: previous cervical treatment 43%, previous preterm birth < 34 weeks 21%, previous mid-trimester loss 26%, uterine anomalies 5%, multiple pregnancy 3%. Spontaneous preterm birth rate < 34 weeks 8%.	Previous preterm birth < 34 weeks. Previous mid-trimester loss. Previous cervical treatment. Uterine anomalies*.* Multiple pregnancy.	*Investigations:* - Transvaginal cervical length ultrasound scans. *Interventions:* - Vaginal progesterone. - Cervical cerclage.	Not reported.
6. **Burul 2014** Retrospective audit (conference abstract only). January 2005 – December 2012. University College London Hospital, London, UK.	125 women; ethnicity and risk factors for spontaneous preterm birth not reported. Spontaneous preterm birth rate not reported, but median gestation at delivery was 35^+ 2^ weeks.	Not reported.	*Investigations:* - Not reported. *Interventions:* - Cervical cerclage.	Not reported.
7. **Grant 2016** Prospective observational study (conference abstract only). January 2014 – January 2016. Royal Derby Hospital, Derby, UK.	146 women; ethnicity not reported. Risk factors for spontaneous preterm birth: previous preterm birth < 37 weeks 36%, previous second trimester miscarriage 13%, previous failed rescue cerclage 1%, previous LLETZ 49%, previous cone biopsy 2%, previous cervical biopsies 2%, medical history (not further defined) 4%. Spontaneous preterm birth rate < 37 weeks 25%.	Previous preterm birth < 37 weeks. Previous second trimester miscarriage. Previous failed rescue cerclage. Previous LLETZ. Previous cone biopsy. Previous cervical biopsies. Medical history (not further defined).	*Investigations:* - Transvaginal cervical length ultrasound scans. *Interventions:* - Vaginal progesterone. - Cervical cerclage.	Not reported.
8. **O’Brien 2010** Qualitative interpretive study. Study dates not reported. Manchester, UK.	14 women; White British 93%, Black Caribbean 7%. Risk factors for spontaneous preterm birth and spontaneous preterm birth rate not reported.	Previous preterm birth. Cervical surgery or other gynaecological procedures that increases the risk of cervical incompetence (not further defined).	*Investigations:* - Transvaginal cervical length ultrasound scans. *Interventions:* - Cervical cerclage. - Vaginal progesterone. - Aspirin. - Antibiotics. - Activity restriction. - Hospital admission.	Timing of planned visit is not reported, however it is noted that women are encouraged to attend as soon as they become pregnant. Frequency of review is weekly, fortnightly or monthly depending on individual needs. Timing of last visit is not reported.
9. **Turitz 2016** Cross-sectional study. November 2009 – June 2013. Hospital of the University of Pennsylvania, Pennsylvania, United States of America.	218 women; African American 83%, Caucasian 12%, other 6%. Risk factors for spontaneous preterm birth: previous second trimester loss 39%, previous spontaneous preterm birth < 37 weeks 71%. Spontaneous preterm birth rate < 37 weeks 36%.	Previous spontaneous preterm birth < 37 weeks. Previous second trimester loss 16–24 weeks.	*Investigations:* - Transvaginal cervical length ultrasound scans. *Interventions:* - IM 17OHP-C for all. - Cervical cerclage also recommended if cervical length ≤ 15 mm or previous preterm birth < 34 weeks.	Not reported.
10. **Manuck 2011** Retrospective cohort study. Usual care patients June 2002 – June 2010, preterm birth clinic patients 2008–2010. Utah, United States of America.	70 preterm birth clinic patients; Caucasian 83%. (153 usual-care patients). Risk factors for spontaneous preterm birth: previous spontaneous preterm birth < 35 weeks 100%. Spontaneous preterm birth rate < 37 weeks 49% in preterm birth clinic patients (63% in usual care patients).	Previous spontaneous preterm birth < 35 weeks.	*Investigations:* - Transvaginal cervical length ultrasound scans. - Vaginal swab for bacterial vaginosis. - Urine culture. - fFN only if symptoms. *Interventions:* - IM 17OHP-C for all. - Cervical cerclage if cervical length < 25 mm at < 22 weeks. If cervical length shortening is detected > 22 weeks: - Hospital admission. - Activity restriction. - Tocolysis (indomethacin) if contracting. - Betamethasone.	The first visit is planned for 10–18 weeks. Frequency of review is six weekly with additional visits as clinically indicated (every 1–2 weeks if the cervix shortens). Women are discharged at 28–32 weeks.
11. **Hughes 2017** Retrospective audit. 2004–2013. Royal Women’s Hospital, Melbourne, Australia.	756 women; ethnicity not reported. Risk factors for spontaneous preterm birth: previous spontaneous preterm birth 54%, previous cervical surgery 24%, uterine malformations 11%, incidental finding of short cervix 9%. Spontaneous preterm birth rate < 37 weeks 21%.	Previous spontaneous preterm birth. Previous mid-trimester loss. Previous cervical surgery: ≥1 cold knife cone biopsy or ≥ 2 LLETZ. ≥3 surgical terminations of pregnancy or ≥ 4 dilatation and curettage procedures. Incidental finding of a short cervix < 25 mm on transvaginal scan in the mid-trimester. Uterine malformation.	*Investigations:* - Transvaginal cervical length ultrasound scans. - Cervical swabs for abnormal flora at each visit and for chlamydia at the first visit. - Serum thyroid stimulating hormone and alkaline phosphatase at the first visit. - fFN at the final visit. *Interventions:* Women are offered treatment if cervical length < 25 mm, options include: - Vaginal progesterone. - Cervical cerclage. - Arabin pessary (as part of a study only). Appropriate antimicrobials as indicated.	The first visit is planned for 14 weeks. Women are seen fortnightly. Women are discharged at 26 weeks.
12. **Newnham 2017** Prospective population-based cohort study. 2009 – December 2015 (November 2014 – December 2015 for assessment of preterm birth clinic) Perth, Western Australia.	154 women cared for in the preterm birth clinic, but data on 92 concluded pregnancies reported only (233,527 births in whole statewide cohort); ethnicity not reported. Risk factors for spontaneous preterm birth: previous early preterm birth 67%, recurrent pregnancy losses 26%, previous cone biopsy or other ablative procedures of the cervix 14%, uterine anomalies 11%, autoimmune conditions 11%, placental risk factors 10%. Preterm birth < 37 weeks 32% (spontaneous preterm birth rate not reported separately).	Previous early preterm birth. Recurrent pregnancy loss. Previous cone biopsy or other ablative procedure of the cervix. Uterine anomalies. Previous stillbirth or neonatal death. Autoimmune conditions. Placental risk factors.	*Investigations:* - Transvaginal cervical length ultrasound scans. *Interventions:* - Vaginal progesterone. - Cervical cerclage. - Mental health support. - ‘Medical interventions’ not further specified.	Timing of planned visit is not reported, however the median gestational age at first visit was 13^+ 6^ weeks. Frequency of review and timing of last visit is not reported.
13. **Stricker 2016** Retrospective cohort study. October 2008 – December 2014. Marburg, Germany.	106 women; ethnicity not reported. Risk factors for spontaneous preterm birth: previous preterm birth < 37 weeks 33%, previous surgical conisation 19%, previous cervical cerclage for a short cervix 12%, short cervix <3rd centile in current pregnancy 48%. Spontaneous preterm birth rate < 37 weeks 44%, < 34 weeks 28%.	Previous preterm birth or mid-trimester loss at 16–37 weeks. Previous surgical conisation. Previous cerclage for a short cervix. Short cervical length < 3rd centile on transvaginal scan in current pregnancy.	*Investigations:* - Transvaginal cervical length ultrasound scans. *Interventions:* For singleton pregnancies with a short cervix <3rd centile: - Cervical pessary. - Vaginal progesterone. - Cervical cerclage.	Gestation of planned first visit and frequency of review not reported. Women are discharged at 32 weeks.
14. **Danti 2014** Randomised controlled trial. May 2000 – May 2003. Hospital of the University of Brescia and University of Turin, Italy.	87 women; Caucasian 95%, others not reported. Risk factors for spontaneous preterm birth: short cervix ≤25 mm at 24–32 weeks 100%, previous preterm delivery or PPROM 14%, previous mid-trimester miscarriages 3%, uterine anomalies (bifid uterus, uterine septum, myoma) 3%, previous cervical surgery 1%. Spontaneous preterm birth rate < 37 weeks 15%.	Previous preterm labour and/or PPROM. Previous mid-trimester miscarriage. Previous cervical insufficiency*.* Previous cervical surgery. Uterine fibromyoma. Uterine malformations Clinical suspicion of cervical shortening.	*Investigations:* - Transvaginal cervical length ultrasound scans. - Vaginal culture for trichomonas, aerobic and/or anaerobic bacteria, chlamydia. - Rectal samples for beta haemolytic streptococcus. *Interventions:* - Cervical cerclage. - Vaginal progesterone. - Targeted antibiotic therapy for positive cultures. Tocolysis (nifedipine) as the study intervention (compared to placebo).	The first visit is planned for 14 weeks. Frequency of planned review not reported. Women are discharged at 34 weeks.

*PPROM* premature pre-labour rupture of membranes, *LLETZ* large loop excision of the transformation zone, *fFN* fetal fibronectin, *IM* intramuscular, *17OHP-C* 17-alpha hydroxyprogesterone caproate, *UK* United Kingdom

**Table 3:** Characteristics of included studies reporting on multiple clinics

Study details	Characteristics of women cared for in the clinic and spontaneous preterm birth rate	Eligibility criteria for referral to the clinic	Investigations and interventions offered	Timing and frequency of review
1. **Kindinger 2016** Retrospective observational study. January 2004 to January 2014. Queen Charlotte’s Hospital, St Mary’s Hospital and Chelsea and Westminster Hospital; London, UK.	725 women; Caucasian 66%, Black 18%, Asian 16%. Risk factors for spontaneous preterm birth: previous excisional cervical treatment to a depth of ≥12 mm 100% (women with other risk factors specifically excluded from this study). Spontaneous preterm birth rate < 37 weeks 9%, < 34 weeks 2%.	Previous preterm birth < 37 weeks. Previous mid-trimester miscarriage > 13 weeks. Uterine anomaly. Previous excisional cervical treatment to a depth of ≥12 mm (cone biopsy, LLETZ or LEEP).	*Investigations:* - Transvaginal cervical length ultrasound scans. *Interventions:* - Cervical cerclage.	The first visit is planned for 13–16 weeks. Frequency of planned review not reported. Women are discharged at 20–23 weeks.
2. **Watson 2017** Prospective cohort study. April 2012 – November 2016. Guys and St Thomas’ Hospital and University College London Hospital; London, UK.	66 women; White 61%, Black 32%, Asian/Middle-Eastern 8%. Risk factors for spontaneous preterm birth: previous spontaneous preterm birth or late miscarriage 100%. Spontaneous preterm birth rate < 37 weeks 35%.	Previous spontaneous preterm birth < 37 weeks. Previous spontaneous late miscarriage between 14 and 24 weeks.	*Investigations:* - Transvaginal cervical length ultrasound scans. *Interventions:* - Cervical cerclage if cervix < 25 mm. - Vaginal progesterone. - Arabin pessary.	Not reported.
3. **Cohen 2014** Retrospective audit (conference abstract only). January 2013 – May 2014. St Mary’s Hospital and Queen Charlotte’s Hospital; London, UK.	509 women; Caucasian 59%, Afro-carribean 15%. Risk factors for spontaneous preterm birth: previous preterm labour < 34 weeks 26%, previous mid-trimester miscarriage 17%, previous excisional cervical treatment 50%, uterine anomalies 2%, multiple pregnancy 3%. Spontaneous preterm birth rate < 37 weeks 11%, < 34 weeks 4%.	Previous preterm labour < 34 weeks. Previous mid-trimester miscarriage. Previous excisional cervical treatment. Uterine anomalies. Multiple pregnancy.	*Investigations:* - Transvaginal cervical length ultrasound scans. *Interventions:* - Cervical cerclage. - Vaginal progesterone.	Not reported.
4. **Kuhrt 2016** Prospective observational study. October 2010 – July 2014. St Thomas’ Hospital, Queen Charlotte’s Hospital, University College London Hospital, West Middlesex University Hospital; London, UK; Manchester St Mary’s Hospital; Manchester, UK.	1249 women. Ethnicity: White 56%, Black 29%, Asian 8%, other 9%. Risk factors for spontaneous preterm birth: previous spontaneous preterm birth 38%, previous PPROM 19%, previous late miscarriage 22%, previous cervical surgery 44%, short cervix < 25 mm 15%. Spontaneous preterm birth rate < 37 weeks 15%, < 34 weeks 8%.	Previous spontaneous preterm birth < 37 weeks. Previous PPROM < 37 weeks. Previous late miscarriage 16–24 weeks. Previous cervical surgery. Cervical length < 25 mm in the current pregnancy.	*Investigations:* - Transvaginal cervical length ultrasound scans. - Quantitative fFN. *Interventions:* - Cervical cerclage: history-indicated if ≥3 late miscarriages or previous spontaneous preterm births < 34 weeks; ultrasound-indicated if cervix < 25 mm. - Vaginal progesterone.	Gestation of planned first visit not reported. Women seen every 2–4 weeks. Women are discharged at 30 weeks.
5. **Vousden 2015** Prospective observational study. November 2010 – July 2014. Fifteen hospitals across the UK, nine of which have preterm birth clinics – St Thomas’ Hospital, Queen Charlotte’s Hospital, University College London Hospital, West Middlesex University Hospital; London; Royal Infirmary of Edinburgh, Edinburgh; Sunderland Royal Hospital; Sunderland; Manchester St Mary’s Hospital; Manchester; University Hospital; Coventry; Royal Victoria Infirmary; Newcastle.	54 women; Black 46%, White 35%, other 19%. Risk factors for spontaneous preterm birth: previous preterm birth 44%, previous second trimester miscarriage 72%, previous cervical surgery 7%. Spontaneous preterm birth rate < 34 weeks 11%.	Previous preterm birth. Previous second trimester miscarriage. Previous cervical surgery.	*Investigations:* - Transvaginal cervical length ultrasound scans. *Interventions:* - Cervical cerclage. - Vaginal progesterone.	Not reported.
6. **Care 2019** Cross-sectional study (survey). March 2017 – July 2017. Thirty-three unnamed clinics across the UK (list obtained from authors but not included here). *Primary and secondary outcome data also obtained from Sharp 2014, which was the original study updated by Care 2019.*	This study reports on the typical practice of preterm birth clinics, not on individual women cared for in them.	*Percentage of clinics with each referral criteria (n = 32 clinics):* Previous preterm birth, 100%, at gestations of: < 37 weeks 13%. < 35 weeks 3%. < 34 weeks 65%. < 32 weeks 13%. < 28 weeks 3%. Other 3%. Previous PPROM, 91%, at gestations of: < 37 weeks 16%. < 34 weeks 55%. < 32 weeks 13%. < 28 weeks 6%. Other 10%. Recurrent second trimester loss 91%. Previous cervical surgery: ≥1 LLETZ 47%. ≥2 LLETZ 100%. Cone biopsy 100%. Uterine anomalies 75%. Recurrent first trimester loss 16%. Threatened preterm labour 13%. Incidental finding of a short cervix 88%.	*Investigations (n = 32 clinics):* - Transvaginal cervical length ultrasound scans 100%. *Additional Investigations (n = 22 clinics from Sharp 2014, not reported in Care 2019):* - Vaginal flora swabs 59%. - Vaginal acidity 0%. - Cervical stress test 14%. - fFN 32%. *Interventions (n = 32 clinics):* Primary treatment choice for asymptomatic women with a short cervix on ultrasound: - Cervical cerclage 30%. - Vaginal progesterone 18%. - IM progesterone 0%. - Arabin cervical pessary 3%. - Combination treatment (most commonly cervical cerclage and vaginal progesterone) 18%. - Multiple first line treatment options 30%. Treatment threshold: - < 25 mm 55%. - < 15 mm 3%. - Centile charts 15%. - Centile chart and/or < 25 mm 12%. - Other cervical length cutoff 3%. QUIPP App 12%. *Additional advice (n = 22 clinics from Sharp 2014, not reported in Care 2019):* - Restricting physical activity 45%. - Sick leave 27%. - Refraining from sexual intercourse 41%. - Nutrition 27%. - Bed rest 0%. - No further advice 36%.	*Gestation of planned first visit (n = 32 clinics):* - < 12 weeks 9%. - 12–14 weeks 38%. - 15–16 weeks 50%. - > 16 weeks 3%. - As soon as referred 0%. *Frequency of planned review (n = 22 clinics from Sharp 2014, not reported in Care 2019):* - Every 2 weeks 18%. - Every 4 weeks 5%. - Based on clinical findings 77%. *Gestation of planned last visit after a diagnosis of short cervix (n = 22 clinics from Sharp 2014, not reported in Care 2019):* - 24 weeks 5%. - 28 weeks 41%. - 30 weeks 5%. - 34 weeks 36% - 37 weeks or delivery 14%.

*PPROM* premature pre-labour rupture of membranes, *LLETZ* large loop excision of the transformation zone, *LEEP* loop electrosurgical excision procedure, *fFN* fetal fibronectin, *IM* intramuscular, *UK* United Kingdom

### Synthesis of results

Data from 39 clinics were combined to assess the primary and secondary outcomes; 33 UK based clinics from Care 2019^23^ and six clinics from individual clinic studies outside of the UK [7, 49–53]. Outcome data was incomplete for some clinics, thus the number of clinics assessed for each outcome varies.

Preterm birth clinic referral criteria is described in Table 4. All clinics accepted referrals for women with a previous spontaneous preterm birth, however the gestation of previous preterm birth varied. Just over half (20/38, 53%) set a threshold of < 34 weeks for review. Previous late miscarriage or mid-trimester loss was the second most common referral criteria reported in 34/38 (89%) clinics. Most clinics also accepted referrals for women with previous cervical surgery (33/38, 87%), although there was variation in the type of surgery and numbers of excisional biopsies required (Table 4).

**Table 4:** Preterm birth clinic referral criteria

Referral criteria (non-exclusive)	Number of clinics (%) *n* = 38 ^a^
Previous spontaneous preterm birth	38 (100)
< 37 weeks	6 (16)
< 35 weeks	2 (5)
< 34 weeks	20 (53)
< 32 weeks	4 (11)
< 28 weeks	1 (3)
Other	1 (3)
No gestational limit reported	4 (11)
Previous late miscarriage/mid-trimester loss	34 (89) ^b^
≥ 16 weeks	2 (5)
No gestational limit reported	32 (84)
Previous PPROM	30 (79)
< 37 weeks	5 (13)
< 34 weeks	17 (45)
< 32 weeks	4 (11)
< 28 weeks	2 (5)
Other	1 (3)
No gestational limit reported	1 (3)
Previous cervical surgery (non-exclusive)	33 (87)
1 LLETZ or no number stated	16 (42)
≥ 2 LLETZ	32 (84)
Knife cone biopsy	33 (87)
Not further defined	1 (3)
Other gynaecological procedures (non-exclusive)	1 (3)
≥ 3 Surgical termination of pregnancy	1 (3)
≥ 4 Dilatation and curettage	1 (3)
Previous cervical cerclage	1 (3)
Uterine abnormality/malformation	27 (71)
Short cervix in current pregnancy	31 (82)
< 25 mm	1 (3)
< 3rd centile for gestation	1 (3)
‘Short’ cervix not further defined	29 (76)
Follow up for threatened preterm labour	4 (11)
Previous stillbirth or neonatal death	1 (3)
Autoimmune conditions	1 (3)
Placental risk factors	1 (3)
Multiple pregnancy	0 (0)

*UK* United Kingdom, *PPROM* preterm pre-labour rupture of membranes, *LLETZ* large loop excision of the transformation zone

^a^ Data not available for one clinic

^b^ Includes 29 clinics who accepted referrals for recurrent second trimester miscarriage as referral for a single second trimester miscarriage not reported in Care 2019

Data on the types of investigations offered were available for 28 clinics (22 UK, 6 non-UK). All clinics performed transvaginal cervical length ultrasound scans, however use of additional investigations was variable. Urogenital swabs were the second most common investigation performed, with 16/28 (57%) clinics routinely offering this. Fetal fibronectin was used as a risk assessment tool in asymptomatic women in some clinics (8/28, 29%). Other investigations included urine culture, rectal culture for *Group B streptococcus*, serum thyroid stimulating hormone and alkaline phosphatase which were each described in one clinic.

There were differences in how interventions aimed at reducing the risk of spontaneous preterm birth were reported. Table 5 lists the range of interventions offered for the six clinics outside of the UK, and separately describes the primary treatment choice for a sonographic short cervix for the 33 clinics within the UK where this information was available. Data on the range of interventions offered in UK clinics was not available for synthesis. Cervical cerclage was offered in all clinics outside of the UK (6/6, 100%). Progesterone was also offered in all clinics, as vaginal progesterone in 4/6 (67%) and intramuscular 17-alpha hydroxyprogesterone caproate (17OHP-C) in the remaining two clinics, both of which were in America. Within UK based preterm birth clinics, the primary treatment choice for women with a sonographic short cervix was cervical cerclage in 10/33 clinics (30%), vaginal progesterone in 6/33 (18%) and cervical pessary in 1/33 (3%). An additional 10/33 clinics (30%) reported utilisation of multiple first-line treatment options, and 6/33 (18%) used a combination of treatment, usually cervical cerclage and vaginal progesterone.

**Table 5:** Preterm birth clinic interventions

Interventions routinely offered (non-exclusive)	Number of non-UK based clinics (%), *n* = 6
Cervical cerclage	6 (100)
Vaginal progesterone	4 (67)
IM progesterone (17OHP-C)	2 (33)
Cervical pessary	1 (17)
Primary choice of intervention for women with a sonographic short cervix ^a^	Number of UK based clinics (%), *n* = 33
Cervical cerclage	10 (30)
Vaginal progesterone	6 (18)
Cervical pessary	1 (3)
Combination therapy ^b^	6 (18)
Multiple first-line treatment options	10 (30)

*UK* United Kingdom, *IM* intramuscular, *17OHP-C* 17-alpha hydroxyprogesterone caproate.

^a^ Threshold for a short cervix defined by the individual clinic, further detailed in the text

^b^ Most were combination of cervical cerclage and vaginal progesterone

Various measures were used to define the threshold for treatment of a ‘short’ cervix. The most common threshold was a cervical length of < 25 mm (21/38, 53%). A cervical length of < 15 mm or use of centile charts were used less frequently (2/38, 5% and 6/38, 16% respectively). A further 4/38 clinics (11%) used a combination of thresholds with centile charts and/or a cervical length of < 25 mm. Results from the QUiPP App, which combines clinical history, cervical length measurements and fetal fibronectin [55] were used by 4/38 (11%) clinics to determine the need for treatment for a short cervix. One clinic reported using an ‘other’ threshold and data were unavailable for another.

The use of additional interventions such as hospital admission, antenatal corticosteroid therapy and antimicrobials for high risk, asymptomatic women was not consistently reported across studies and these data were not available from the large surveys of practice in the UK; thus accurate synthesis of information was not possible. Data on additional interventions are provided in Tables 2 and 3 where this was reported in individual studies.

Many clinics also provided routine lifestyle recommendations. Of the 22 clinics (all in the UK), where these data were available, almost half (10/22, 45%) routinely advised restriction of physical activity, 6/22 (27%) recommended stopping work, 9/22 (41%) advised refraining from sexual intercourse and 6/22 (27%) made dietary recommendations. No clinic recommended routine bed rest and 8/22 (36%) clinics reported that no additional advice was given.

Table 6 describes the planned timing of preterm birth clinic visits. Data were available for the planned first appointment for 35 clinics (32 UK, 3 non-UK), and for planned last appointment for 26 clinics (22 UK, 4 non-UK). Most clinics planned to see women for their first appointment at 12 to 14 weeks (14/35, 40%) or 15 to 16 weeks (16/35, 46%). The timing of discharge from a preterm birth clinic varied considerably from 24 to 37 weeks. The planned frequency of review was available for 24 clinics (22 UK, 2 non-UK) with the majority (18/24, 75%) individualising this depending on clinical findings. Five clinics (21%) reviewed women fortnightly, and one clinic (4%) four-weekly.

**Table 6:** Timing of planned first and last preterm birth clinic appointments

Gestational age at planned first clinic appointment	Number (%), *n* = 35 ^a^
< 12 weeks	3 (9)
12–14 weeks	14 (40)
15–16 weeks	16 (46)
> 16 weeks	1 (3)
Other ^b^	1 (3)
Gestational age at planned last clinic appointment ^c^	Number (%), *n* = 26 ^d^
24 weeks	1 (4)
26 weeks	1 (4)
28 weeks	9 (35)
30 weeks	1 (4)
32 weeks	2 (8)
34 weeks	9 (35)
37 weeks or delivery	3 (12)

*UK* United Kingdom

^a^ Data not available for four clinics

^b^ 10–18 weeks

^c^ If a gestational age range was given, the upper gestation is reported

^d^ Data not available for 13 clinics

## Discussion

### Summary of evidence

Data was obtained for a number of preterm birth clinics in this systematic review. The majority of clinics were located in the UK, but clinics in America, Germany, Italy and Australia are also identified. All clinics accepted referrals for women with a previous spontaneous preterm birth, however other referral criteria varied. The majority of clinics saw women with previous mid-trimester loss, previous preterm pre-labour rupture of membranes, previous cervical surgery, uterine abnormality or malformation, and a short cervix detected in pregnancy. A minority of clinics also accepted referrals for other indications including history of multiple surgical terminations of pregnancy or dilatation and curettage, as follow up after a diagnosis of threatened preterm labour, and presence of an autoimmune condition. Of interest, no clinic listed previous caesarean section at full dilatation as a referral criteria, despite recent evidence that this is a significant risk factor for spontaneous preterm birth [54, 57].

Transvaginal cervical length scans were used to aid decisions on management in all clinics but the use of additional investigations such as urogenital swabs, urine culture and fetal fibronectin varied. Differences in how interventions were reported limited the ability to synthesise these results, however available evidence shows that a range of interventions are available, with significant variation in the choice of primary management for a sonographic short cervix within the UK, where this data was available. The majority of clinics saw women for their first appointment between 12 and 16 weeks of gestation and cared for them through to the late second or early third trimester. The frequency of preterm birth clinic review was usually directed by clinical findings.

### Application of results

To the best of our knowledge, this is the first systematic review to assess practice in preterm birth clinics globally, and has shown wide variation in most aspects of care. Inconsistencies in care have also been identified as an issue in national surveys of practice in the UK [23, 40]. This information can be used to support the development and implementation of preterm birth clinic consensus guidelines and national prevention programmes, which are likely to improve consistency and encourage best practice care based on current evidence. The newly introduced ‘Reducing Preterm Birth: Guidelines for Commissioners and Providers’ [13] is likely to fulfil this role in the UK and may also influence care in other countries; re-evaluation of practice following implementation of this guideline will be important to assess its impact. The findings from this review can also be used to assist with service planning as preterm birth clinics continue to be introduced throughout the developed world.

Improving consistency in care will also allow clinics to combine their outcome data in a more meaningful way, enabling high-quality research into the effectiveness of interventions provided in preterm birth clinics, along with comparisons between clinics [5]. The UK Preterm Clinical Network has already developed a bespoke internet-based database that uses an agreed minimal dataset, allowing systematic and standardised collection of clinical data from preterm birth clinics within the network [58]. In 2018, there were seven sites using this database, and an additional 24 sites were registered as Data Collection Centres, four of which are outside of the UK [58]. This collaborative approach to data collection, if combined with a consistent approach to care in preterm birth clinics, has great potential for the future evaluation of existing and new interventions aimed at optimising the care of asymptomatic women at high risk of spontaneous preterm birth.

### Limitations

The main limitation of this review is the potential for incomplete data. Due to the paucity of literature in this area, studies were included that did not specifically assess or report on care in a preterm birth clinic, but reported on another aspect of care in a group of high risk women cared for in a preterm birth clinic. Thus, details about the clinic itself were at times incomplete. We have assumed for the purposes of this review that if a referral criteria, investigation or intervention was not reported, then it was not used. Another limitation is that included data is predominantly from clinics in the UK, so results are likely to favour practice from this region. This is unsurprising as the UK have led the development of modern-day preterm birth clinics and to our knowledge, are the first to recommend the use of preterm birth clinics in national guidelines [13]. Results from the UK also have a lower risk of publication bias due to the availability and inclusion of studies that had taken a cross-sectional survey approach to assessing preterm birth clinic practice [23, 40]. Results from outside of the UK may reflect care from academic preterm birth clinics which are more likely to have published their data or be involved in other research. National or binational surveys of practice in other localities would be helpful and we intend to explore this in Australasia.

We have taken a unique approach to analysis by combining different studies on the same clinic and in our selection of studies suitable for combination and synthesis. This was necessary as the ‘population’ of interest was the clinic itself, and thus inclusion of all studies would have resulted in over-representation of certain clinics which were described in multiple studies. The assumption that the three clinics reported in other included UK based studies were included within the four anonymous clinics in Care 2019, is a further limitation of this study. However even if not the case, this is unlikely to have changed findings significantly.

## Conclusions

To our knowledge, this is the first systematic review of the practice of preterm birth clinics internationally. Variation in the referral criteria, investigations and interventions, and timing and frequency of review in individual preterm birth clinics was evident. Consistency in care is likely to be improved with the introduction of consensus guidelines and national preterm birth prevention programmes such as those recently introduced in the UK. A repeat survey of practice in preterm birth clinics in the UK can be used to assess the impact of new consensus guidelines introduced in the UK, and are also required in other localities.

## Supplementary information

**Additional file 1: Table S1.** MEDLINE search strategy. **Table S2.** Methodological quality assessment of included studies based on the Newcastle-Ottawa Scale for cohort and case controlled studies. **Table S3.** Methodological quality assessment of included studies based on the modified Newcastle-Ottawa Scale for cross-sectional studies. **Table S4.** Methodological quality assessment of included studies based on the Cochrane Risk of Bias Tool for randomized controlled trials. **Table S5.** Methodological quality assessment of included qualitative studies based on the Critical Appraisal Skills Programme Checklist for qualitative research.

## Data Availability

All data generated or analysed during this study are included in this published article and its supplementary information files.

## Abbreviations

CASP: Critical Appraisal Skills Programme
MeSH: Medical Subject Headings
NICE: National Institute for Health and Care Excellence
PRESS: Peer Review of Electronic Search Strategies
PRISMA: Preferred Reporting Items for Systematic Reviews and Meta-Analyses
PROSPERO: International Prospective Register of Systematic Reviews
UK: United Kingdom
