# Supplementary material for: The use of specialised preterm birth clinics for women at high risk of spontaneous preterm birth: a systematic review
Source: BMC Pregnancy Childbirth. 2020 Jan 29;20:58. doi: 10.1186/s12884-020-2731-7 (PMC6990596; doi:10.1186/s12884-020-2731-7)
Supplement: Supplementary file 1 — Additional file 1: Table S1. MEDLINE search strategy. Table S2. Methodological quality assessment of included studies based on the Newcastle-Ottawa Scale for cohort and case controlled studies. Table S3. Methodological quality assessment of included studies based on the modified Newcastle-Ottawa Scale for cross-sectional studies. Table S4. Methodological quality assessment of included studies based on the Cochrane Risk of Bias Tool for randomized controlled trials. Table S5. Methodological quality assessment of included qualitative studies based on the Critical Appraisal Skills Programme Checklist for qualitative research. [file 12884_2020_2731_MOESM1_ESM.docx]

**Supplementary Table 1. MEDLINE search strategy**

| **Line** | **Search term** | **Results** |
| --- | --- | --- |
| 1 | ((preterm or pre-term) adj3 (clinic or clinics)).mp | 46 |
| 2 | (miscarriage adj2 (followup or follow-up) adj2 (clinic or clinics)).mp | 0 |
| 3 | ((multidisciplinary or multi-disciplinary) adj2 antenatal adj2 (clinic or clinics)).mp | 7 |
| 4 | (special* adj (antenatal or ante-natal) adj (clinic or clinics)).mp | 40 |
| 5 | ((special* adj2 (clinic or clinics)) and (preterm or pre-term)).mp | 35 |
| 6 | ((special* adj2 (clinic or clinics)) and (premature adj (delivery or parturition or birth* or labo?r))).mp | 16 |
| 7 | 1 or 2 or 3 or 4 or 5 or 6 | 115 |
| 8 | Prenatal Care/og [Organization & Administration] | 1427 |
| 9 | Prenatal Care/ and (Ambulatory Care Facilities/ or Outpatient Clinics, Hospital/) | 217 |
| 10 | Perinatal Care/ | 4153 |
| 11 | Pregnancy/ and (Ambulatory Care Facilities/ or Outpatient Clinics, Hospital/) | 1567 |
| 12 | 8 or 9 or 10 or 11 | 7070 |
| 13 | Pregnancy, High-Risk/ | 4586 |
| 14 | Premature Birth/ | 12141 |
| 15 | Obstetric Labor, Premature/ | 13071 |
| 16 | Pregnancy Complications/ | 87239 |
| 17 | 13 or 14 or 15 or 16 | 111873 |
| 18 | 12 and 17 | 1037 |
| 19 | exp animals/ not humans.sh | 4574521 |
| 20 | (7 or 18) not 19 | 1142 |
| 21 | Limit 20 to (english language and yr =”1998-Current”) | 887 |

***** This is a truncation symbol on MEDLINE and was used to retrieve terms with a common root.

**Supplementary Table 2. Methodological quality assessment of included studies based on the Newcastle-Ottawa Scale for cohort and case controlled studies**

| **Study** | **Selection** | | | | **Comparability** | **Outcome** | | | **Overall Score**  (out of 9) |
| --- | --- | --- | --- | --- | --- | --- | --- | --- | --- |
|  | **Representa-tivness (⋆)** | **Selection of non-exposed cohort (⋆)** | **Ascertainment of exposure (⋆)** | **Outcome not present at start (⋆)** | **By design or control of confounders (⋆⋆)** | **Assessment (⋆)** | **Length of follow-up (⋆)** | **Adequacy of follow-up (⋆)** |  |
| Bolt 2011 ^a^ | **⋆** | **-** | **⋆** | **⋆** | **- -** | **⋆** | **⋆** | **⋆** | 6 |
| Ivandic 2018 ^a^ | **⋆** | **-** | **⋆** | **⋆** | **- -** | **⋆** | **⋆** | **⋆** | 6 |
| Karkhanis 2012 ^a^ | **⋆** | **-** | **⋆** | **⋆** | **- -** | **⋆** | **⋆** | **-** | 5 |
| Yulia 2015 ^a^ | **⋆** | **-** | **⋆** | **⋆** | **- -** | **⋆** | **⋆** | **-** | 5 |
| Kindinger 2013 ^a^ | **⋆** | **-** | **⋆** | **⋆** | **- -** | **⋆** | **⋆** | **-** | 5 |
| Burul 2014 ^a^ | **⋆** | **⋆** | **⋆** | **⋆** | **- -** | **⋆** | **⋆** | **-** | 6 |
| Grant 2016 ^a^ | **⋆** | **-** | **⋆** | **⋆** | **- -** | **⋆** | **⋆** | ***** | 6 |
| Manuck 2011 | **⋆** | **⋆** | **⋆** | **⋆** | **- ⋆** | **⋆** | **⋆** | - | 7 |
| Hughes 2017 ^a^ | **⋆** | **-** | **⋆** | **⋆** | **- -** | **⋆** | **⋆** | ***** | 6 |
| Newnham 2017 | **⋆** | **⋆** | **⋆** | **⋆** | - - | **⋆** | **-** | **⋆** | 6 |
| Stricker 2016 | **⋆** | - | **⋆** | **⋆** | - - | **⋆** | **⋆** | **⋆** | 6 |
| Kindinger 2016 ^a^ | **-** | - | **⋆** | **⋆** | - - | **⋆** | **⋆** | **⋆** | 5 |
| Watson 2017 | **⋆** | **⋆** | **⋆** | **⋆** | - - | **⋆** | **⋆** | **⋆** | 7 |
| Cohen 2014 ^a^ | **⋆** | - | **⋆** | **⋆** | - - | **⋆** | **⋆** | **-** | 5 |
| Kuhrt 2016 ^a^ | **⋆** | - | **⋆** | **⋆** | - - | **⋆** | **⋆** | **⋆** | 6 |
| Vousden 2015 ^a^ | **-** | - | **⋆** | **⋆** | - - | **⋆** | **⋆** | **⋆** | 5 |

^a^ Observational study, but not a typical cohort or case controlled study.

**Supplementary Table 3. Methodological quality assessment of included studies based on the modified Newcastle-Ottawa Scale for cross-sectional studies**

| **Study** | **Selection** | | | | **Comparability** | **Outcome** | | **Overall Score**  (out of 10) |
| --- | --- | --- | --- | --- | --- | --- | --- | --- |
|  | **Representa-tivness (⋆)** | **Sample size (⋆)** | **Non-respondents (⋆)** | **Ascertainment of the exposure (⋆⋆)** | **By design or control of confounders (⋆⋆)** | **Assessment (⋆⋆)** | **Statistical test (⋆)** |  |
| Turitz 2016 | **⋆** | **⋆** | **⋆** | **⋆⋆** | - - | **⋆⋆** | **⋆** | 8 |
| Care 2019 | **⋆** | **⋆** | - | - - | - - | - - | - | 2 |

**Supplementary Table 4. Methodological quality assessment of included studies based on the Cochrane Risk of Bias Tool for randomized controlled trials**

| **Study** | Danti 2014 |
| --- | --- |
| **Random sequence generation (selection bias)** | Low: central computer-generated randomization. |
| **Allocation concealment (selection bias)** | Low: randomized list of balanced blocks for every 10 participants. |
| **Blinding of participants and personnel (performance bias)** | Low: patients and clinicians (except pharmacist) blinded. |
| **Blinding of outcome assessment (detection bias)** | Low: blinding maintained until after delivery of last participant. |
| **Incomplete outcome data (attrition bias)** | Low: outcome data available for all. |
| **Selective outcome reporting (reporting bias)** | High: unplanned secondary analysis for women with a short cervix. |
| **Other bias** | Unclear: early closure, did not reach planned sample size. |

**Supplementary Table 5. Methodological quality assessment of included qualitative studies based on the Critical Appraisal Skills Programme Checklist for qualitative research**

| **Study** | O’Brien 2010 |
| --- | --- |
| **Study aims** | Yes, aims to gain an understanding of the experiences of women attending a preterm birth clinic and to elicit their views. |
| **Appropriate methodology** | Yes, qualitative methodology appropriate for the stated aim. |
| **Study design** | Yes, research design justified, with use of focus groups and interviews. |
| **Recruitment strategy** | Yes, detailed strategy of recruitment from a preterm birth clinic. |
| **Data collection** | Yes, through focus groups and one-to-one interviews with transcription of results. |
| **Researcher-participant relationship** | Unclear, not well described. |
| **Ethical considerations** | Yes, ethical approval obtained. |
| **Data analysis** | Yes, description of thematic analysis by two independent researchers, with categories defined by comments made from the women. |
| **Study findings** | Yes, three main themes were identified and implications of findings discussed. |
| **Value of the study** | Yes, a unique study that identified areas where further research is required. |
